# Biological degradation and mineralization of tetracycline antibiotic using SBR equipped with a vertical axially rotating biological bed (SBR-VARB)

**DOI:** 10.1007/s10532-023-10018-5

**Published:** 2023-02-25

**Authors:** Ali Ahmad Aghapour, Nazila Alizadeh, Hassan Khorsandi

**Affiliations:** Department of Environmental Health Engineering, Urmia University of Medical Sciences, Urmia, Iran

**Keywords:** Tetracycline, Biodegradation, SBR, Vertical axially rotating biological bed, SBR-VARB, Wastewater treatment

## Abstract

Tetracycline (TC) is a widely used antibiotic with a complex aromatic chemical structure and is highly resistant to biodegradation. In this study, an SBR equipped with a vertical axially rotating biological bed (SBR-VARB) was used for the biodegradation and mineralization of TC. SBR-VARB showed high efficiency in removing TC (97%), total phenolic compounds (TP) (95%), and COD (85%) under optimal operating conditions (TC = 50 mg/L, HRT = 1.75 d, and OLR = 36 g COD/m^3^ d). The SBR-VARB was able to treat higher concentrations of TC in shorter HRT than reported in previous studies. The contribution of VARB to improve SBR efficiency in removing TC, TP, and COD was 16, 36, and 48%, respectively. Intermediate compounds formed during the biodegradation of TC were identified using GC–MS under the optimal operating conditions of the bioreactor. These are mainly organic compounds with linear chemical structures. Based on the complete biodegradation of TC under the optimal operating conditions of the bioreactor, 93% and 36% of the chlorine and nitrogen atoms in the chemical structure of TC appeared in the wastewater, respectively. According to the sequence analysis of 16SrDNA, *Pseudomonas* sp., *Kocuria Polaris*, and *Staphylococcus* sp. were identified in the biofilm of VARB and the suspended biomass of the bioreactor. Therefore, SBR-VARB showed high efficiency in the biodegradation and mineralization of TC and can be used as a suitable option for treating wastewater containing antibiotics and other toxic compounds.

## Introduction

Tetracycline (TC) is an antibiotic with a complex and phenolic structure. TC is produced and consumed as the second most widely used antibiotic due to its low production cost and high antimicrobial activity (Chen et al. 2011; Lindsey et al. 2001; Zhang et al. 2011). Due to the high resistance of TC to degradation in the human body, about 70% of it is released undestroyed into wastewater through urine and feces. Due to the high consumption of TC and its low degradation, it can be released into the environment through sewage, agricultural runoff, and even industrial effluents. The concentration of TC in hospital and pharmaceutical wastewater is reported to be 100 to 500 mg/L (Ahmadi et al. 2017).

Introducing TC into the environment may increase the number of antibiotic-resistant bacteria and the risk of infection in humans and animals. TC is a phenolic compound classified as a priority pollutant by the U.S. Environmental Protection Agency (USEPA). The limit for total phenolic compounds for discharge to surface waters is 1 ppb and 1 mg/L for the environment. Therefore, TC must be removed from wastewater before it enters the environment and water resources (Aghapour et al. 2013a, b, 2015; Khorsandi et al. 2018).

Various physical and chemical methods, such as adsorption(Martins et al. 2015; Zhang et al. 2015), chemical oxidation (Gómez-Pacheco et al. 2011; Wu et al. 2010), electrocoagulation (Belkheiri et al. 2011), and combined chemical-biological processes (Ferrag-Siagh et al. 2013; Shi et al. 2011; Xiong et al. 2017; Yahiat et al. 2011), have been investigated for the removal and degradation of TC. There are few studies on the biodegradation of TC using SBR (under anaerobic and anoxic conditions) and the activated sludge process (under anoxic and methanogenic conditions). The mentioned studies reported about 90% and 44% removal efficiencies for SBR and activated sludge process, respectively (Shi et al. 2011; Cetecioglu et al. 2013; Rezaei et al. 2022). Previous studies focused on the biodegradation of TC by anaerobic and anoxic processes. Due to the high growth rate of aerobic microorganisms and the high electrical potential difference between electron receiver and electron donor, aerobic biodegradation processes are preferred over anaerobic and anoxic processes to degrade complex, toxic, and resistant compounds (Tchbanoglous et al. 2003). Previous studies on the biodegradation of TC using bacterial species also indicate that the aerobic process does not produce toxic intermediates (Shao and Wu 2020). Therefore, aerobic processes take precedence over other processes in this regard. Accordingly, the development of aerobic biodegradation processes is essential to remove TC as an antibiotic commonly found in wastewater (Shao and Wu 2020).

Among the aerobic biodegradation processes, sequencing batch reactor (SBR) is widely used for the treatment of industrial wastewater due to its flexibility, operational simplicity, implementation of all stages of wastewater treatment in a tank, resistance to organic shocks, and no need for sludge return (Aghapour et al. 2013a, b, 2015; Khorsandi et al. 2018; Rezaei et al. 2022; Tchbanoglous et al. 2003; Yusoff et al. 2016). However, due to the high TC toxicity as an antibiotic compound, it is necessary to strengthen the SBR by using the attached growth process. Attached growth biomass offers several advantages, including having diverse bacteria groups, resistance to toxic and hydraulic shocks, low energy consumption, and low sludge production (Rezaei et al. 2022; Arya et al. 2016; Bitton 2005; Hosseini and Borghei 2005). When the attached growth biomass is used as a Vertical axially rotating biological bed (VARB) in SBR, it can be more effective for increasing the process efficiency because of the increase in the oxygen and substrate material transfer rate. According to the literature review, no study has investigated TC degradation using an SBR equipped with a VARB. Accordingly, this study aimed to investigate the efficiency of SBR-VARB for the removal of TC and COD from synthetic wastewater.

## Materials and methods

### Synthetic wastewater

Synthetic wastewater was prepared daily by dissolving a determined volume of tetracycline hydrochloride (Sigma-Aldrich ≥ 98%) in tap water. Also, a specific ratio of the nutrient solution was added to the prepared wastewater sample. To prepare the nutrient solution, 5 g K_2_HPO_4_, 15 g KH_2_PO_4_, 120 g NH_4_Cl, 12 g (NH_4_) 2HPO_4_, 10 g CaCO_3,_ and 10 g NaHCO_3_ were added to 1 L of tap water (Aghapour et al. 2013a, b, 2015; Khorsandi et al. 2018; Rezaei et al. 2022). TC was used as a source of carbon and energy, and the nutrient solution was used as a source of nitrogen and phosphorus for the biomass. The COD: N: P ratio in the synthetic wastewater was set to 100: 5: 1 at all experiment stages (Aghapour et al. 2013a, b, 2015; Khorsandi et al. 2018; Rezaei et al. 2022).

### Set up and operation of the bioreactor

**Fig. 1:**
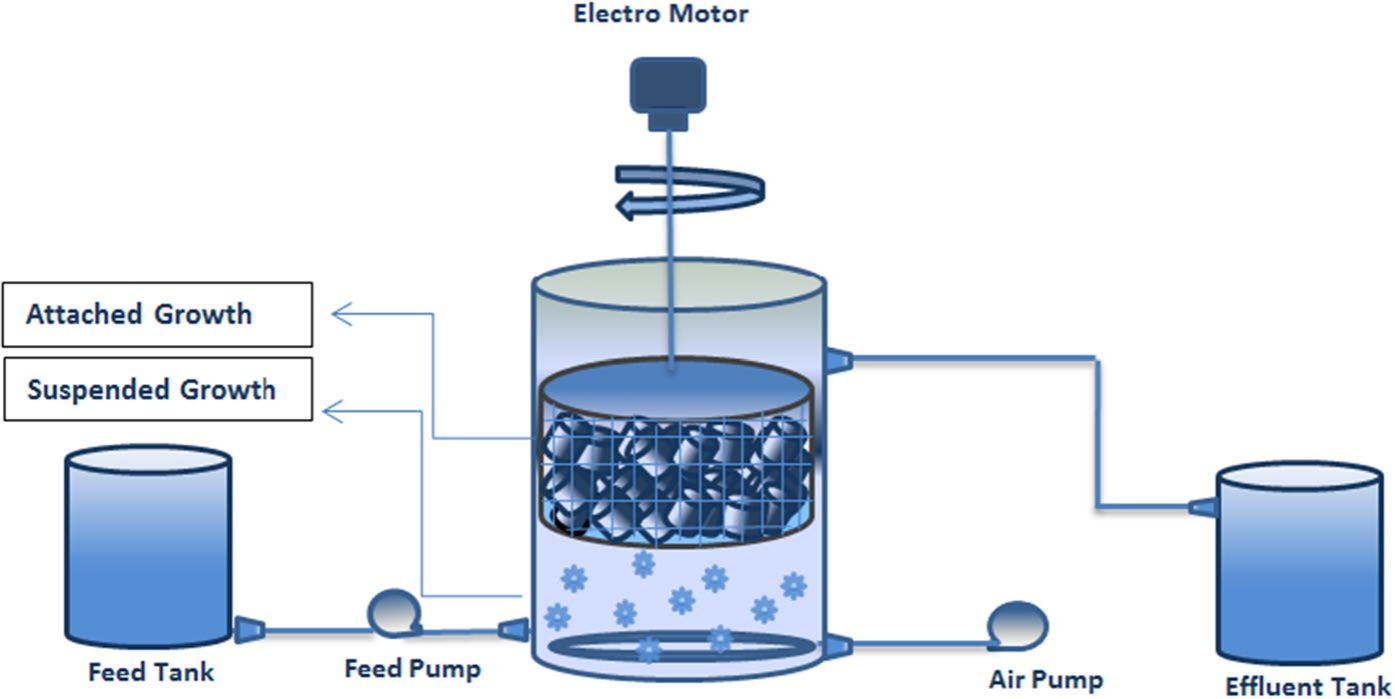
The schematic drawing of the bioreactor

The schematic drawing of SBR-VARB used in this study is shown in Fig. 1. The experiments were conducted in a cylindrical reactor with a practical volume of 7 L and a dimension of 30 cm high and 20 cm in diameter. An air sampling pump (BUYO) was used to supply the airstream the biomass. The vertical axially rotating biological bed occupied 30% of the total volume of the bioreactor and was filled with high-density polyethylene (2HHDPE) cubes (1 cm^3^) packing Media with a specific surface area of 535 m^2^/m^3^, a protected area of 339 m^2^/m^3^, and a density of 109 kg/m^3^ and rotated at a speed of 25 rpm.

In this study, the activated sludge of a municipal wastewater treatment plant with an MLSS concentration of about 5000 mg/L was used to supply the biomass needed for the bioreactor. In all stages of this study, the concentration of MLSS was kept constant in the concentration range of 5000 ± 200 mg/L. Due to the antibiotic effect of TC and its high resistance to biodegradation, biomass acclimation was carried out at a concentration of 1 mg/L. Each operating cycle of the bioreactor has four consecutive stages, including the feeding and reaction stage (2 h), reaction stage (21 h), settling stage (45 min), and discharge stage (15 min). Because of the antibiotic effect of TC and its resistance to biodegradation, the time of the reaction stage is relatively high (21 h).

Table 1 shows the schedule of SBR-VARB operations. As can be seen in Table 1, due to the high toxicity of tetracycline on biomass and its high resistance to biological degradation, a low initial TC concentration of 1 mg/L and a relatively high HRT of 7 days were used for biomass acclimation. TC concentration was increased from 3 to 75 mg/L to investigate the effect of TC concentration on bioreactor efficiency. The optimal TC concentration (50 mg/L) was

**Table 1:** Experimental phases and operation conditions of SBR-VARB

Phases	Step	Day	Inlet TC	Inlet COD (mg/L)	HRT	OLR
			(mg/L)		(d)	(g COD/m3 d)
Acclimation	0	0–40	1	1.7	7	0.18
Effect of concentration	A	41–67	3	5	7	0.53
	B	68–103	5	8.5	7	0.9
	C	104–142	10	17	7	1.8
	D	143–162	15	25.5	7	2.7
	E	163–176	20	34	7	3.6
	F	177–190	30	51	7	5.4
	G	191–206	50	85	7	9
	H	207–222	75	127.5	7	13.5
Effect of HRT	I	251–269	50	85	3.5	18
	J	270–284	50	85	1.75	36
	K	285–298	50	85	1.16	54
Effect of RBP	L	299–325	50	85	1.16	54

used, and the hydraulic retention time was reduced from 7 days to 1.16 d to investigate the effect of hydraulic retention time. The effect of the rotating biological bed on the efficiency of the bioreactor was investigated in the optimal operating conditions (C: 50 mg/L and HRT: 1.75 d).

At each stage of the present study, the operating conditions were changed after the efficiency of the bioreactor reached a steady-state condition. In this study, the steady-state condition was defined as the condition in which the bioreactor efficiency for TC removal is decreased to less than 5% over a week (Aghapour et al. 2013a, b, 2015; Khorsandi et al. 2018).

### Identification of TC degrading microbial species

After biological culture and purification, this study identified the bacterial species using Polymerase Chain Reaction (PCR). The 16 s rRNA gene was extracted and replicated by the method described in previous studies (Chavshin et al. 2012; Weisburg et al. 1991).

### Analysis method

To prepare the samples and determine the values of various parameters in the wastewater, the samples were passed through a paper filter with a 0.45 μm. The samples were taken from the inlet and outlet of the bioreactor. TC concentration was measured using an Agilent HPLC (Rezaei et al. 2022). The HPLC was equipped with a C18 column and UV–visible detector at 275 nm. The mobile phase was oxalic acid/acetonitrile (75:25%) at a 1 mL/min flow rate and 23 °C.

Moreover, an Agilent 7890A Gas Chromatography-Mass Spectrometry (GC–MS) system was used to identify the intermediate compounds derived from the TC degradation during HRT of 6, 12, and 24 h. The GC–MS was equipped with an HP-5 MS column with a length of 30 mm, an inner diameter of 0.25 mm, and a thickness of 0.25 μm. Helium gas with high purity was applied as the carrier gas at a 1 mL/min flow rate. The GC–MS device was operated under the following conditions: EI of 70 eV and injection valve temperature of 250 °C. The Wily 2007 and NIST 2005 libraries were used to identify intermediate compounds in the studied samples (Rezaei et al. 2022; Isarain-Chávez et al. 2010).

Regarding the phenolic structure of TC, the possibility of converting it into other phenolic compounds and total phenolic compounds (TP) in the effluent of the bioreactor were also measured using the standard method D 5220. To determine the removal rate of COD associated with TC, the COD concentration in the effluent of the bioreactor was determined based on the standard method D 5530 (Rezaei et al. 2022). Ammonia, nitrite, nitrate, and chloride levels were measured using standard methods 4500-NH_3_, 4500-NO_2_^−^, 4500-NO_3_^−^, and 4500-CL, respectively. The MLSS concentration was also determined using the standard method 2540 D (Rezaei et al. 2022; Federation and Association 2005).

A scanning electron microscope (Philips and XL30 model) was used to identify the morphology of the biofilm formed on 2H media. To identify the biofilm formed on the media, in the optimal operating conditions of the bioreactor, several 2H media were removed from the bioreactor and dried at 25 °C for 24 h. Then the surfaces of fresh media and media with biofilm were analyzed using SEM.

## Results and discussion

### Acclimation

This study performed biomass acclimation to TC with an initial concentration of 1 mg/L; the results are presented in Fig. 1. The SBR-VARB efficiency increased for TC removal in the first few days, followed by a decreasing trend. The increase in the bioreactor efficiency can be due to the absorption of TC in the biomass, especially in the dead microbial mass that has a higher potential for adsorbing TC than the living microbial mass. The higher potential of deadly microbial mass is related to the rupture of the cytoplasmic membrane of microorganisms (Ferrag-Siagh et al. 2013; Shi et al. 2011; Rezaei et al. 2022; Li and Zhang 2010). After several days, the SBR-VARB efficiency reduced and showed significant fluctuations, while on days between 29 and 35, its efficiency reached a steady-state condition. Considering the biodegradation of over 95% of TC and 85% of TP in the bioreactor, it can be concluded that the bioreactor was wholly acclimated to TC, and it can biodegrade TC and use it as the only carbon source. Generally, the time required to acclimate the biomass to toxic compounds under aerobic conditions varies from several hours to several days, depending on the type and concentration of toxic substances (Aghapour et al. 2013a, b, 2015; Khorsandi et al. 2018; Rezaei et al. 2022). In previous studies, it has been reported that about one month is required for the biomass to acclimate to TC (Khorsandi et al. 2018; Cetecioglu et al. 2013). During the acclimation, microorganism species capable of producing CT-degrading enzymes (e.g., Glutathione S-Transferase (GSTs) and Laccase enzymes) can grow and reproduce (Park 2012; Park and Choung 2007).

### Effect of inlet TC Concentration

**Fig. 2:**
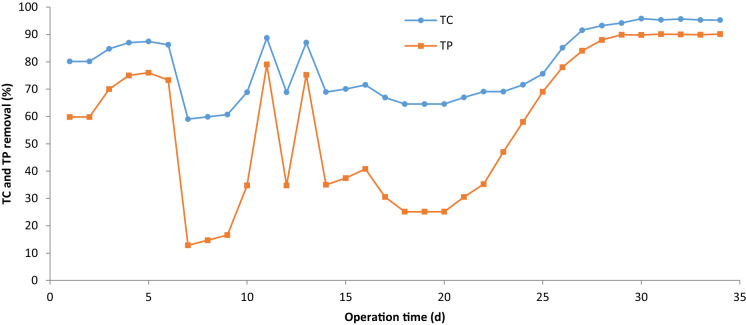
TC and TP removal during the acclimation phase

The concentrations of TC in the inlet flow of the SBR-VARB were selected at 3, 5, 10, 15, 20, 30, 50, and 75 mg/L, depending on the ability of the bioreactor for the biodegradation of the pollutants. These results are shown in Fig. 2. As observed here, increasing the concentration of TC in each stage decreased the bioreactor efficiency for TC removal in the first few days. It then reached a steady-state condition after various fluctuations. Reducing the bioreactor efficacy due to the increased concentrations of TC can be associated with increased toxicity and the bactericidal effect of TC at higher concentrations.

The TC biodegradation is accomplished by the detoxification enzymes produced by microorganisms, including Glutathione S-Transferase and Laccase enzymes, which convert toxic compounds into non-toxic compounds for microorganisms (Park 2012; Park and Choung 2007). Based on measuring the concentrations of enzymes in biological processes, the level of enzymes increased with the concentration of toxic compounds in the bioreactor (Aghapour et al. 2013a, b, 2015; Khorsandi et al. 2018; Rezaei et al. 2022). Hence, the production of the detoxification enzymes increased with increasing the TC concentration, which increased the bioreactor efficiency. Despite the temporary reduction caused by increasing pollutant concentrations early, the process efficiency generally increased. In similar studies on the biodegradation of amoxicillin using the SBR, it has also been reported that with increasing the antibiotic concentration, the SBR efficiency temporarily reduced and then increased (Aghapour et al. 2013a, b, 2015; Khorsandi et al. 2018; Polesel et al. 2016). Therefore, the results of this study are consistent with the findings of previous studies.

**Fig. 3:**
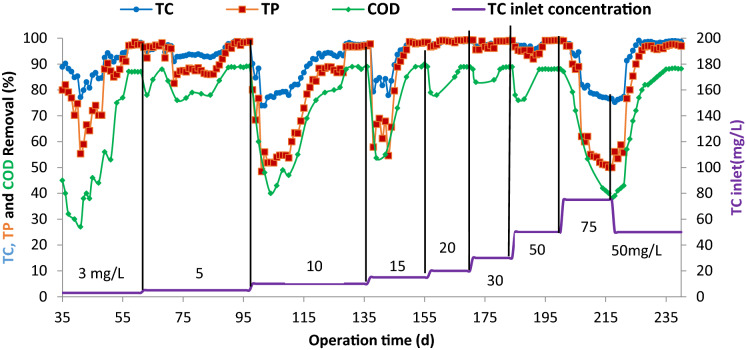
Profile the removal efficiencies of TC, TP, and COD at various inlet concentrations

As observed in Fig. 3, by increasing the concentrations of TC up to 50 mg/L, the bioreactor quickly recovered its ability to degrade TC. Consequently, up to a concentration of 50 mg/L, TC was used by the biomass as the sole source of carbon (Wiggins et al. 1987). However, with increasing concentration of TC from 50 to 75 mg/L, bioreactor efficiency showed a progressive reduction (50 to 99%). Due to the inhibitory effect of TC, its toxic effect prevails at high concentrations and inhibits the growth of microorganisms (Wiggins et al. 1987). Therefore, at a concentration of 75 mg/L, TC is considered a toxic substance and cannot be used as a carbon source for biomass (Khorsandi et al. 2018). The maximum acceptable TC concentration for SBR-VARB was 50 mg/L in this research.

**Fig. 4:**
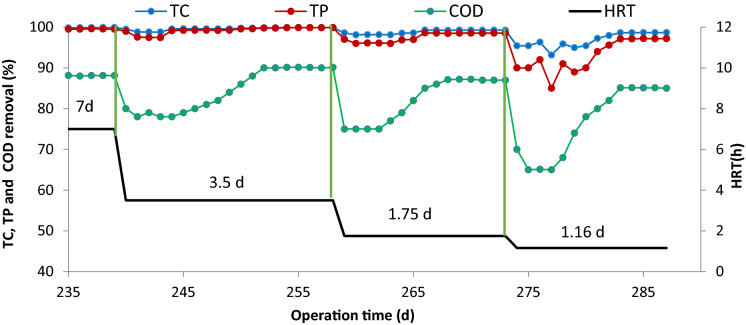
Profile of TC, TP, and COD removal efficiencies at various HRT

The average bioreactor efficiencies in removing TC and TP at different TC concentrations of 3, 5, 10, 15, 20, 30, and 50 mg/L were determined to be above 97% under steady-state conditions. Also, the average bioreactor efficiencies for COD removal at different studied concentrations (3, 5, 10, 15, 20, 30, and 50 mg/L) were above 85% under steady-state conditions. Because of the similar efficiency of the bioreactor in the removal of TC and COD, it can be concluded that the biological process completely degraded the TC antibiotic. As shown in Fig. 4, the removal efficiencies of TC and TP were almost similar, while a slightly lower efficiency was observed for the COD removal. It can be due to the remaining tiny amount of the intermediate compounds (presented in Table 2) produced from the biodegradation of TC (Aghapour et al. 2013b, 2015).

**Table 2:** Partial 16S rRNA sequence of isolated bacteria

Name	partial sequence of 16S ribosomal RNA gene
*Pseudomonas* sp.	GCAGTCGCAGCGCGATGCAGAGGAGCTTGCTCC TCGATTCAGCGGCGGACGGGTGAGTAATGCCTAGGAATCTGCCTAGTAGT GGGGGACAACGTTTCGAAAGGAACGCTAATACCGCATACGTCCTACGGGA GAAAGTGGGGGATCTTCGGACCTCACGCTATTAGATGAGCCTAGGTCGGA TTAGCTAGTTGGTAGGGTAAAGGCCTACCAAGGCGACGATCCGTAACTGG TCTGAGAGGATGATCAGTCACACTGGAACTGAGACACGGTCCAGACTCCT ACGGGAGGCAGCAGTGGGGAATATTGGACAATGGGCGAAAGCCTGATCCA GCCATGCCGCGTGTGTGAAGAAGGCCCTCGGGTCGTAAAGCACTTTAAGT TGGGAGGAAGGGCTTACAGCGAATACCTGTGAGTTTTGACGTTACCGACA GAATAAGCACCGGCTAACTTCGTGCCAGCAGCCGCGGTAATACGAAGGGT GCAAGCGTTAATCGGAATTACTGGGCGTAAAGCGCGCGTAGGTGGCTTGA TAAGTTGGATGTGAAATCCCCGGGCTCAACCTGGGAACTGCATCCAAAAC TGTCTGGCTAGAGTGCGGTAGAGGGTAGTGGAATTTCCAGTGTAGCGGTG AAATGCGTAGATATTGGAAGGAACACCAGTGGCGAAGGCGACTACCTGGA CTGACACTGACACTGAGGTGCGAAAGCGTGGGGAGCAAACAGGATTAGAT ACCCTGGTAGTCCACGCCGTAAACGATGTCAACTAGCCGTTGGGATCCTT GAGATCTTAGTGGCGCAGCTAACGCATTAAGTTGACCGCCTGGGGAGTAC GGCCGCAAGGTTAAAACTCAAATGAATTGACGGGGGCCCGCACAAGCGGT GGAGCATGTGGTTTAATTCGAAGCAACGCGAAGAACCTTACCTGGCCTTG ACATGCTGAGAACTTTCCAGAGATGGATTGGTGCCTTCGGGAACTCAGAC ACAGGTGCTGCATGGCTGTCGTCAGCTCGTGTCGTGAGATGTTGGGTTAA GTCCCGTAACGAGCGCAACCCTTGTCCTTAGTTACCAGCACATTATGGTG GGCACTCTAAGGAGACTGCCGGTGACAAACCGGAGGAAGGTGGGGATGAC
*Kocuria polaris*	TTACCNTGCAAGTCGACGATGATGCCCAGCTT GCTGGGCGGATTAGTGGCGAACGGGTGAGTAATACGTGAGTAACCTGCCC TTGACTCTGGGATAAGCCTGGGAAACTGGGTCTAATACTGGATACTACCG TCCACCGCATGGTGGGTGGTGGAAAGGGTTTTACTGGTTTTGGATGGGCT CACGGCCTATCAGCTTGTTGGTGGGGTAATGGCTCACCAAGGCGACGACG GGTAGCCGGCCTGAGAGGGTGACCGGCCACACTGGGACTGAGACACGGCC CAGACTCCTACGGGAGGCAGCAGTGGGGAATATTGCACAATGGGCGGAAG CCTGATGCAGCGACGCCGCGTGAGGGATGACGGCCTTCGGGTTGTAAACC TCTTTCAGTAGGGAAGAAGCGAGAGTGACGGTACCTGCAGAAGAAGCGCC GGCTAACTACGTGCCAGCAGCCGCGGTAATACGTAGGGCGCAAGCGTTGT CCGGAATTATTGGGCGTAAAGAGCTCGTAGGCGGTTTGTCGCGTCTGCTG TGAAAGCCCGGGGCTCAACCCCGGGTCTGCAGTGGGTACGGGCAGACTAG AGTGCAGTAGGGGAGACTGGAATTCCTGGTGTAGCGGTGAAATGCGCAGA TATCAGGAGGAACACCGATGGCGAAGGCAGGTCTCTGGGCTGTTACTGAC GCTGAGGAGCGAAAGCATGGGGAGCGAACAGGATTAGATACCCTGGTAGT CCATGCCGTAAACGTTGGGCACTAGGTGTGGGGGACATTCCACGTTTTCC GCGCCGTAGCTAACGCATTAAGTGCCCCGCCTGGGGAGTACGGCCGCAAG GCTAAAACTCAAAGGAATTGACGGGGGCCCGCACAAGCGGCGGAGCATGC GGATTAATTCGATGCAACGCGAAGAACCTTACCAAGGCTTGACATTCACC GGACCGCCCCAGAGATGGGGTTTCCCTTCGGGGCTGGTGGACAGGTGGTG CATGGTTGTCGTCAGCTCGTGTCGTGAGATGTTGGGGTTAAGTCCCGCAA CGAGCGCAACCCTCGTTCTATGTTGCCAGCACGTGATGGTGGGGGACTCA TAGGAAGACTGCCCGGGGTCAACTCGGAAGGAAGGTGGGGGATGACGTCA AATCATCATGCCCCCTTATGTCTTGGGGCTTCACGCATGGCTACAATGGG CC
*Staphylococcus* sp.	ATTGCATGTCGAGCGAACAGAGAAGGAG CTTGCTCCTTTGACGTTAGCGGCGGACGGGTGAGTAACACGGGGGTAACC TACCTATAAAACTGGGATAACTTCGGGGGGGGGGAGCTAATACCGGGTAA TATTTGGAACCGGATGGTTCTATAGTGAAAAATGGTTTTGCTATGGCTTG TAGATGGAGCCGCGCGGTGTTATCTAGGTGGAAAGGTAACGGCTTACCAA GGCGACGATACGGGGAGGACCTGAGAGGGTGATCGGGCAGGGTGGGAGGG GGGGCGGACGACACTCCTACGGGAGGCAGCAATAGGGAATCTTCCGCAAG GGGCTAGAGCCTGACGGAGCAACGCCGCGTGAGTGATGAAGGTCTTCTGA TCGTAAAACTCTGTTATTGGGGAACAACATGGGTGTAGGTAAGTATGCAC GTGTTGACGGGACCTAATGAGAGAACCACGGCTAGCTACATGGCACCGGC CGCGGTAATACGTAGGTGGCAAGCGTTATCCGGAATTATTGGGCGTAAAG CGCGCGTAGGCGGTTTTTTAAGTCGGATGTGAAAGCCCACGGCTCAACCG TGGAGGATCATTGGAAACTGGAAAACTTGAGTGCAGAAGAGGAAAGTGGA ATTCCATGTGTAGCGGTGAAATGCGCAAAGATATGGAGGAACACCAGTGG CGAAGGCGACTTTCTGGTCTGTAACTGACGCTGATGTGCGAAAGCGTGGG GATCAAACAGGATTAGATACCCTGGTAGTCCACGCCGTAAACGATGAGTG CTAAGTGTTAGGGGGTTTCCGCCCCTTAGTGCTGCAGCTAACGCATTAAG CACTCCGCCTGGGGGAGTACNACCGCAAGGTTTGAAACTCAAAGGAATTG ACGGGGACCCGCACAAGCGGTGGAGCATGTGGGTTTAATTCTAATCTACG CGAAAACCTTACCAATCTTGACATCTTTGACAACTCTAAAGATAAAGCTT TCCCCTTCGGGGACAAAGTGACAGTGGTGCATGGTTGTCGTCAGCTCGTG TCCGTGAGAATGATTGGGATTAGTCCCGCAACGAACGCCAACCCTTAGCC TTAATTGCCCATCACTAAGATGCGGCACTCTTAGTTGGACTTGCCGGGTG ACAAACCGAC

Previous studies on the biodegradation of TC have mainly focused on using anaerobic and anoxic processes using a co-substrate Cetecioglu et al. 2013. The highest concentration of TC in anaerobic biological processes has been 8.5 mg/L. While in this study, the SBR-VARB was capable of treating an initial TC concentration of 50 mg/L. It could be related to the high reaction rate of aerobic processes compared with anaerobic processes. Due to the high electrical potential difference between the electron receiver (O_2_) and electron donor (organic matter) in aerobic processes, the reaction rate in aerobic processes is higher than in the anaerobic processes. On the other hand, using co-substrates in biological processes increases the operation cost of these processes (Khorsandi et al. 2018). Therefore, the SBR-VARB is a cost-effective process due to the use of TC as the sole carbon source compared to other methods using co-metabolism to degrade such pollutants processes.

In a previous study, the aerobic biodegradation process was applied for TC removal using sequencing batch biofilm reactors (SBBRs) with a concentration of 50 μg/L (Matos et al., 2014). The results indicated that the aerobic biodegradation process had a suitable efficiency for TC removal. Therefore, the SBR-VARB can accept higher concentrations of TC than SBBRs.

### Effect of hydraulic retention time (HRT)

**Fig. 5:**
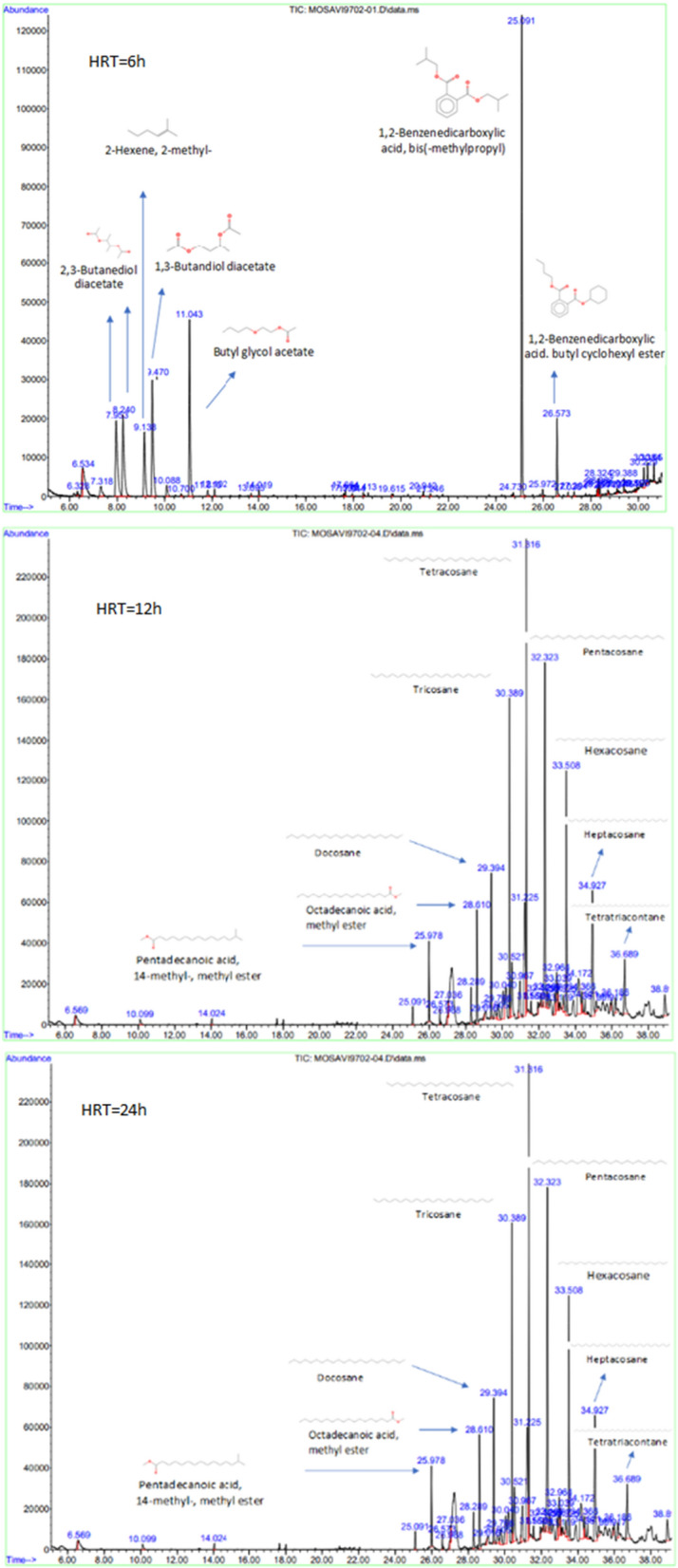
The main intermediate compounds resulting from the biodegradation of TC

Hydraulic Retention Time (HRT) is one of the most critical parameters in designing and estimating the construction costs of wastewater treatment plants. In this study, after selecting the optimal concentration of the target pollutant (50 mg/L), the effect of HRT was investigated on the bioreactor performance. In this regard, HRT decreased from 7 d to 3.5, 1.75, and 1.16 d. Figure 5 illustrates the effect of reducing HRT on the efficiency of the bioreactor in the removal of TC and COD. As shown in Fig. 5, by reducing the HRT, the bioreactor efficiency was decreased by removing TC and COD for several days. This reduction in the bioreactor efficiency for COD removal is higher than for TC removal (Aghapour et al. 2013a, b, 2015; Khorsandi et al. 2018; Rezaei et al. 2022). These conditions cause incomplete biodegradation of TC and are partly converted into intermediate compounds (Table 2). So, the bioreactor efficiency showed a further reduction for COD removal. This finding can be attributed to the decreased HRT, increased organic loading rate (OLR) from 9 to 18, 36, and 54 gCOD/m^3^, and limited bioreactor capacity (Aghapour et al. 2013a, b, 2015; Khorsandi et al. 2018). However, the bioreactor efficiency increased with increasing contact time and reached a steady-state condition after several days. In the steady-state condition, the average removal efficiencies of TC at HRTs of 7, 3.5, 1.75, and 1.16 d were 99, 99, 98, and 97%, respectively. Also, the average removal efficiencies of COD were 90, 90, 87, and 85% at the mentioned HRTs, respectively. Therefore, even at an HRT of 1.16 h, the bioreactor showed high efficiency for the biodegradation and mineralization of TC. This result confirmed the increased capacity of the bioreactor for the treatment of wastewater with high OLR of toxic compounds.

Also, the comparison of the results presented in Figs. 2 and 3 showed that the increased TC concentration had a more significant effect on the bioreactor efficiency than that of HRT. So, the OLR can be increased up to 9 gCOD/m^3^.d by increasing the TC concentration. While by reducing HRT (or increasing the inlet flow into the bioreactor), the process efficiency increased to 56 gCOD/m^3^. Also, by lowering the HRT, the shocks caused by changing the operating conditions in the bioreactor were smaller than the shock caused by the increase in the TC concentration in the first few days. Therefore, the effect of TC concentration on the bioreactor efficiency was much higher than that of the HRT. It can be attributed to the high toxicity of the TC compound at high concentrations.

The compliance of the effluent quality of the bioreactor with environmental standards is one of the main criteria for assessing bioreactors used for purifying toxic wastewater (Aghapour et al. 2013a, b, 2015; Khorsandi et al. 2018). There is no recommended maximum acceptable concentration for TC in the environmental standards. However, due to the phenolic structure of medicine, it is necessary to consider a recommended limit for its concentration in the bioreactor effluents (1 mg/L) (Aghapour et al. 2013a, b, 2015; Khorsandi et al. 2018). In the present research, the concentration of TP residues in the bioreactor effluent at HRT of 7, 3.5, 1.75, and 1.16 d were 0.48, 0.51, 0.82, and 1.12 mg/L, respectively. Therefore, the TP concentration at HRTs of ≤ 1.75 was higher than the recommended standards for discharging effluent into the environment (1 mg/L). As a result, the optimal operation conditions of this bioreactor included an initial TP concentration of 50 mg/L and an HRT of 1.75 d.

### Identification of main intermediates and biodegradation pathway of TC

The main intermediate compounds resulting from the biological degradation of TC were identified using GC–MS, and the mass spectra of intermediates at HRT of 6, 12and 24 h are shown in (Fig. 5Rezaei et al. 2022). As can be seen in Fig. 5, the main intermediate compounds resulting from biological degradation during the hydraulic retention time of 6 h include the cyclic compounds such as 1,2-Benzenedicarboxylic acid, bis(2-methylpropyl) ester, 1,2-Benzenedicarboxylic acid, butyl cyclohexyl ester linear compounds such as 2,3-Butanedioldiacetate, 2-Hexene, 2-methyl-, 1,3-Butandiol diacetate, Butyl glycol acetate. The primary intermediates obtained during the HRT of 6 h are not hydrogenated and nitrogenous compounds. Therefore, dehalogenation and removing amino groups and nitrogen atoms from TC in the first 6 h is almost complete.

In the complete biodegradation of TC under stoichiometric conditions, 3.69 mg/L of chlorine is expected to be released into the water. In this study, about 3.43 mg/L of chlorine was added to the chlorine concentration of water. Thus, about 93% of chlorine is dehalogenated and released into the water. The release of chlorine atoms has not been reported in previous studies on the biodegradation of TC. However, the dechlorination of 2,4,6-trichlorophenol has been about 90% (Khorsandi et al. 2018a).

The concentrations of ammonia, nitrite, and nitrate in the bioreactor effluent were 0.041, 0.002, and 1.021 mg/L-N, respectively, under optimal conditions. Since about 5.86% of the molecular weight of the TC (C_22_H_25_ClN_2_O_8_:480.9 g/mol) is made up of nitrogen atoms, the total concentration of nitrogen compounds is estimated to be about 2.95 mg/L-N in the complete biodegradation under stoichiometric conditions. Due to the absence of intermediate compounds containing nitrogen atoms in the effluent, it can be deduced that a significant amount of the nitrogen atoms in TC was first converted to ammonia and then to nitrate through nitrification. Finally, the nitrate was converted to N_2_ by denitrification in the sedimentation stage of the operation cycle of the bioreactor and then removed from the wastewater. Therefore, in this study, the sum concentration of ammonia (0.041 mg/L-N), nitrite (0.002 mg/L-N), and nitrate (1.021 mg/L-N) in the effluent was 1.063 mg/L-N. Therefore, about 36% of zinc atoms are released into the bioreactor effluent. Simultaneous nitrification and denitrification have been reported in previous studies due to the presence of aerated and non-aerated phases, such as sedimentation, in the SBR operation cycle (Li et al. 2019). Identification of *Staphylococcus* sp. as a nitrifying bacterium and *Pseudomonas* sp. as a denitrifying bacterium can also confirm the biological transformations of nitrogenous compounds (Deng et al. 2021).

Also, as can be seen in Fig. 5, the main intermediate compounds resulting from the biological degradation of TC become simpler compounds with increasing HRT so that at HRT of 24 h, all major intermediate compounds, including Pentadecanoic acid, 14-methyl-, methyl ester, Octadecanoic acid, methyl ester, Docosane, Tricosane Tetratriacontane, Pentacosane, Hexacosane, Heptacosane, Tetratriacontane, are linear compounds.

### The effect of vertical axially rotating biological bed on SBR

**Fig. 6:**
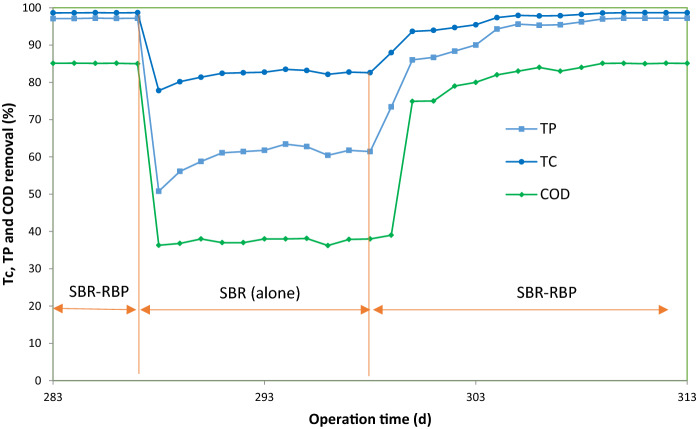
Profile of TC, TP, and COD removal efficiencies in SBR-VARB and SBR

Because of the equipping of SBR with VARB, it is necessary to examine the effect of the VARB on the overall performance of SBR-VARB. To determine the contribution of VARB to the overall efficiency of SBR-VARB, the performance of a single SBR was investigated under optimal conditions. The results are shown in Fig. 6. As observed here, by removing VARB from the bioreactor, its efficiency for removing TC, TP, and COD decreased sharply from 97, 95, and 85% to 82, 61, and 38%, respectively. Therefore, the contribution of VARB to the removal of TC, TP, and COD was 16, 36, and 48%, respectively. To investigate the ability of combined SBR-VARB to remove the target pollutants, VARB was re-installed in the SBR reactor and used for TC removal. According to the results, the bioreactor efficiency enhanced and decreased after 15 d. Over time, the bioreactor efficiency gradually reaches the previous performance for the biodegradation and mineralization of TC. As a result, the role of VARB is significant in increasing the efficiency of the SBR-VARB. The role of VARB in increasing the efficiency of SBR can be attributed to the following reasons: First, adding the attached growth biomass (biofilm) has a synergistic effect with suspended growth biomass in SBR-VARB. Due to more diverse bacterial species, high resistance to toxic and hydraulic shocks, and low energy consumption, the attached growth biomass is more efficient than suspended growth biomass in the biodegradation of toxic compounds. Hence, due to the high resistance of the attached growth biomass to hydraulic shock and toxicity, it can reduce the high concentrations of toxic substances in the feeding phase and subsequently reduce the toxicity of wastewater (Aghapour et al. 2013b, 2015; Moussavi and Heidarizad 2010, 2011; Moussavi et al. 2009). Accordingly, the conditions are more suitable for the activity of suspended growth biomass, and the efficiency of the bioreactor is increased due to a higher rate of biodegradation reaction. Therefore, the performance of SBR-VARB increased as a result of the collaboration of the attached growth biomass and suspension growth biomass. This synergic effect is significant when the attached growth biomass is formed as the biofilm with a low thickness (Aghapour et al. 2013b, 2015; Moussavi and Heidarizad 2010, 2011). The second reason for the increase in mass transfer is attributed to the rotation of VARB inside the SBR. If the biomass is used in a rotating state in the bioreactor, the attached growth biomass is formed as a biofilm on the media. Due to the low thickness of biofilm and rotation of media in the bioreactor, the transfer rates of oxygen and substrate from wastewater to biofilm and waste materials produced by the biofilm from the biofilm to the wastewater are much more than that in attached-growth and suspended-growth processes (Aghapour et al. 2013b, 2015; Moussavi and Heidarizad 2011; Moussavi et al. 2009; Leili and Moussavi 2014). Given the advantages mentioned above, VARB can dramatically increase the efficiency of the SBR.

### *Identification of biofilm morphology formed on media in* SBR-VARB

**Fig. 7:**
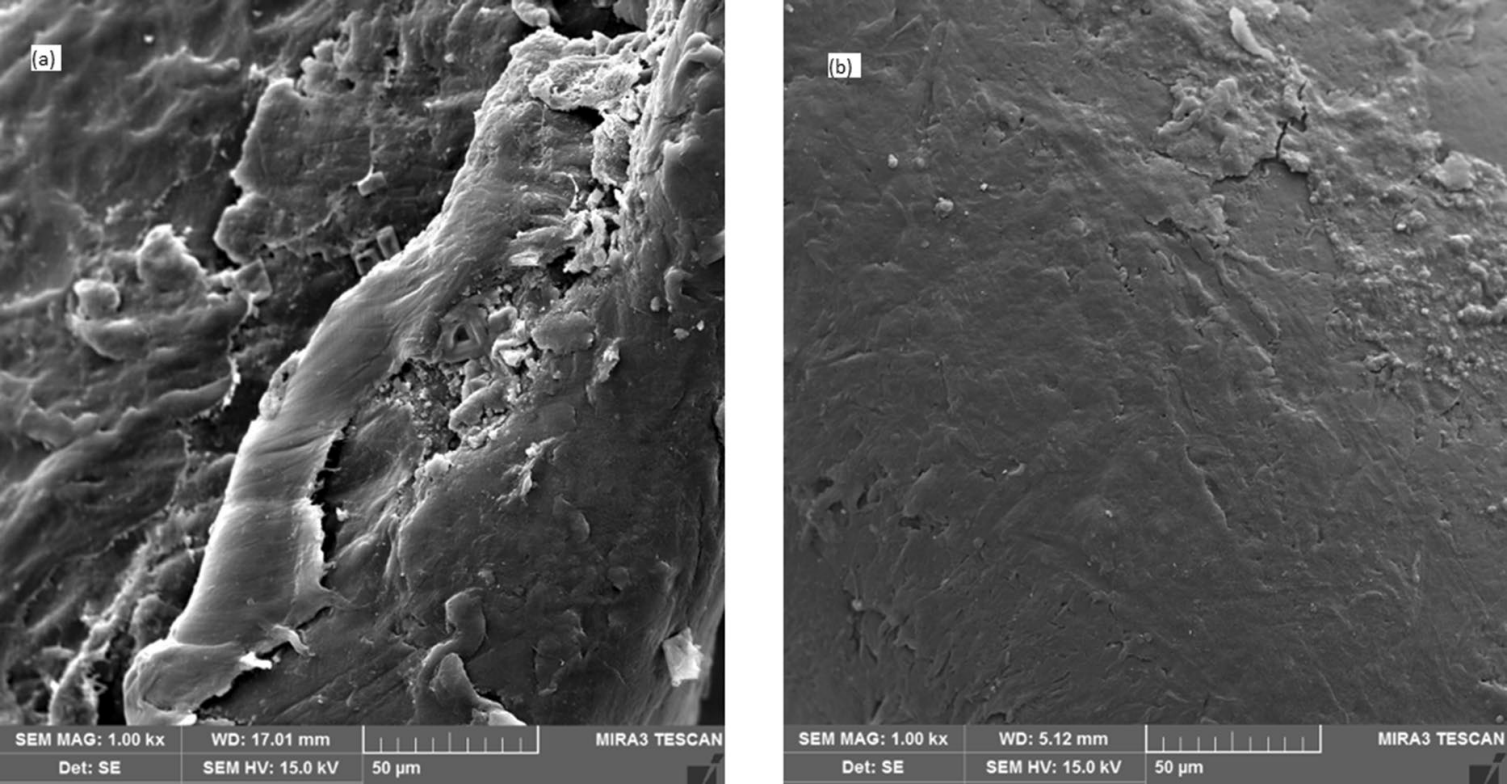
The SEM image of fresh **a** used 2H media in SBR-VARB **b**

Scanning Electron Microscopy (SEM) was used to identify the biofilm formed on the media, and its results are shown in Fig. 7a, b. Figure 7a shows that the surface of fresh 2H media is not smooth, with many heterogeneous pores. However, the surface of the 2H media used in the bioreactor (Fig. 7b) has become almost smooth due to the adhesion of the biological agents in the bioreactor and its rotation. The biofilm formed on the media is visible and soft, which has been scratched due to contact with other media.

### Isolation and Identification of TC hydrochloride biodegrading Bacterium

For the first time in this study, *Pseudomonas* sp., *Kocuria Polaris,* and *Staphylococcus* sp. were isolated from the biofilm of VARB and suspended biomass of the bioreactor and identified according to the sequence analysis of 16S rDNA. Their partial 16S rRNA sequences are presented in Table 2.

*Pseudomonas* sp. was isolated from the biofilm attached to the VARB media, and *Kocuria Polaris* and *Staphylococcus* sp. were also isolated from the suspended biomass in the bioreactor. *Pseudomonas* sp., as an aerobic, heterotrophic, and Gram-negative bacteria, has been used in several previous studies for the biodegradation of various pollutants, such as TC (Wu et al. 2019), 2,4-dichlorophenoxyacetic acid (Karami et al. 2016), 2, 4-nitritolone (Karami et al. 2016), o-nitrobenzaldehyde (ONBA)(Fang-Bo et al., 2006), 2,4-dinitrotoluene(Suen and Spain 1993), Aniline(Anson and Mackinnon 1984), and malachite green (Tao et al. 2017). Although this bacterium has been used to biodegrade various compounds, it was nevertheless identified as the TC biodegrading bacterium for the first time. *Pseudomonas sp.* can also be considered a denitrifier in anaerobic conditions, such as the settling step, which converts and removes the nitrate produced to N_2_ through denitrification(Deng et al. 2021).

*Staphylococcus* sp., as an optional anaerobic, heterotrophic, and Gram-positive bacterium, is classified in the nitrifying bacteria group. This bacterium is also capable of degrading several antibiotics (e.g., TC, sulfathiazole, ampicillin) (Park and Choung 2007) and aromatic compounds (e.g., benzene, biphenyl, naphthalene, 2,4-dichlorophenoxyacetic acid) (Karami et al. 2016). *Staphylococcus* sp. and *Pseudomonas* sp. can produce glutathione S-transferases (GSTs) as a family of detoxification enzymes (Al-Mohaimeed et al. 2022). So, they are capable of biodegradation of many aromatic compounds. However, this bacterial species has not been reported in previous studies on the biodegradation of TC.

One of the reasons for the growth of *Staphylococcus* sp. as a nitrifying bacterium in the SBR-VARB is the presence of the Amino group in the chemical structure of TC and releasing them into the wastewater through the biodegradation process. At the beginning of the TC biodegradation, the Amino groups of the TC appeared as ammonia in the wastewater and then converted to nitrite and nitrate through the nitrification process. Therefore, suitable environmental conditions are provided for the growth of strains of nitrifying bacteria.

Moreover, *Kocuria Polaris* as aerobic, heterotrophic, and Gram-positive bacteria are classified in the *actinobacteria* group. As Phosphors-Accumulating Organisms (PAOs), these bacteria play an essential role in phosphorus removal from wastewater (Larsen et al. 2006). Due to the prevailing anaerobic conditions at the reaction phase of SBR-VARB in the operation cycle and prevailing anaerobic conditions at the sedimentation phases, there is an appropriate condition in the SBR-VARB for the growth of PAOs. However, these bacteria have not yet been used for the biodegradation of TC, but a previous study has evaluated their ability to biodegrade pentachlorophenol (Karn et al. 2011). Therefore, this study identified four bacterial species capable of biodegrading TC for the first time. However, other TC biodegrading bacterial species, such as *Schwarzschild sp*., *Bacillus sp*., and *Pseudomonas* sp., were previously identified (Shao and Wu 2020).

## Conclusion

In this study, SBR-VARB was used for the biodegradation and mineralization of TC. SBR-VARB was able to treat higher concentrations of TC (50 mg/L) in shorter HRT (1.75 d) than reported in previous studies. The results suggest that VARB played an important role in improving SBR performance. The intermediates formed during the biodegradation of TC were linear compounds. In this study, the new TC -biodegrading bacteria were isolated from the biofilm and biomass of SBR-VARB. Therefore, SBR-VARB has high efficiency in the biodegradation and mineralization of TC and can be used as a suitable option for treating wastewater containing antibiotics and other toxic compounds.

## References

[CR1] Aghapour AA, Moussavi G, Yaghmaeian K (2013). Biological degradation of catechol in wastewater using the sequencing continuous-inflow reactor (SCR). J Environ Health Sci.

[CR2] Aghapour AA, Moussavi G, Yaghmaeian K (2013). Investigating the performance of a novel cyclic rotating-bed biological reactor compared with a sequencing continuous-inflow reactor for biodegradation of catechol in wastewater. Bioresour Technol.

[CR3] Aghapour AA, Moussavi G, Yaghmaeian K (2015). Degradation and COD removal of catechol in wastewater using the catalytic ozonation process combined with the cyclic rotating-bed biological reactor. J Environ Manage.

[CR4] Ahmadi M, Ramezani Motlagh H, Jaafarzadeh N, Mostoufi A, Saeedi R, Barzegar G (2017). Enhanced photocatalytic degradation of tetracycline and real pharmaceutical wastewater using MWCNT/TiO2 nano-composite. J Environ Manage.

[CR5] Al-Mohaimeed AM, Abbasi AM, Ali MA, Dhas DSD (2022). Reduction of multiple antibiotics from the waste water using coated glutathione S-transferase producing biocatalyst. Environ Res.

[CR6] Anson JG, Mackinnon G (1984). Novel Pseudomonas plasmid involved in aniline degradation. Appl Environ Microbiol.

[CR7] Arya V, Philip L, Murty Bhallamudi S (2016). Performance of suspended and attached growth bioreactors for the removal of cationic and anionic pharmaceuticals. Chem Eng J.

[CR8] Belkheiri D, Fourcade F, Geneste F, Floner D, Aït-Amar H, Amrane A (2011). Feasibility of an electrochemical pre-treatment prior to a biological treatment for tetracycline removal. Sep Purif Technol.

[CR9] Bitton G (2005). Wastewater microbiology.

[CR10] Cetecioglu Z, Ince B, Gros M, Rodriguez-Mozaz S, Barceló D, Orhon D (2013). Chronic impact of tetracycline on the biodegradation of an organic substrate mixture under anaerobic conditions. Water Res.

[CR11] Chavshin AR, Oshaghi MA, Vatandoost H, Pourmand MR, Raeisi A, Enayati AA (2012). Identification of bacterial microflora in the midgut of the larvae and adult of wild caught Anopheles stephensi: a step toward finding suitable paratransgenesis candidates. Acta Trop.

[CR12] Chen G, Zhao L, Dong Y-h (2011). Oxidative degradation kinetics and products of chlortetracycline by manganese dioxide. J Hazard Mater.

[CR13] Deng M, Zhao X, Senbati Y, Song K, He X (2021). Nitrogen removal by heterotrophic nitrifying and aerobic denitrifying bacterium *Pseudomonas* sp DM02 Removal performance, mechanism and immobilized application for real aquaculture wastewater treatment. Bioresour Technol.

[CR14] Federation WE, Association A (2005). Standard methods for examining water and wastewater.

[CR15] Ferrag-Siagh F, Fourcade F, Soutrel I, Aït-Amar H, Djelal H, Amrane A (2013). Tetracycline degradation and mineralization by the coupling of an electro-Fenton pretreatment and a biological process. J Chem Technol Biotechnol.

[CR16] Gómez-Pacheco CV, Sánchez-Polo M, Rivera-Utrilla J, López-Peñalver J (2011). Tetracycline removal from waters by integrated technologies based on ozonation and biodegradation. Chem Eng J.

[CR17] Hosseini S, Borghei S (2005). The treatment of phenolic wastewater using a moving bed bio-reactor. Process Biochem.

[CR18] Isarain-Chávez E, Arias C, Cabot PL, Centellas F, Rodríguez RM, Garrido JA (2010). Mineralization of the drug β-blocker atenolol by electro-Fenton and photoelectro-Fenton using an air-diffusion cathode for H2O2 electrogeneration combined with a carbon-felt cathode for Fe2+ regeneration. Appl Catal B.

[CR19] Karami S, Maleki A, Karimi E, Poormazaheri H, Zandi S, Davari B (2016). Biodegradation of 2,4-dichlorophenoxyacetic acid by bacteria with highly antibiotic-resistant pattern isolated from wheat field soils in Kurdistan. Iran Environ Monit Assess.

[CR20] Karn SK, Chakrabarti SK, Reddy MS (2011). Degradation of pentachlorophenol by *Kocuria* sp. CL2 isolated from secondary sludge of pulp and paper mill. Biodegradation.

[CR21] Khorsandi H, Ghochlavi N, Aghapour AA (2018). Biological degradation of 2,4,6-trichlorophenol by a sequencing batch reactor. Environ Process.

[CR22] Larsen P, Eriksen PS, Lou MA, Thomsen TR, Kong YH, Nielsen JL (2006). Floc-forming properties of polyphosphate accumulating organisms in activated sludge. Water Sci Technol.

[CR23] Leili M, Moussavi G (2014). Removal of furfural from wastewater using Combined Catalytic Ozonation Process (COP) and Cyclic Biological Reactor (CBR). Avicenna J Environ Health Eng.

[CR24] Li B, Zhang T (2010). Biodegradation and adsorption of antibiotics in the activated sludge process. Environ Sci Technol.

[CR25] Li C, Liu S, Ma T, Zheng M, Ni J (2019). Simultaneous nitrification, denitrification, and phosphorus removal in a sequencing batch reactor (SBR) under low temperature. Chemosphere.

[CR26] Lindsey ME, Meyer M, Thurman EM (2001). Analysis of trace levels of sulfonamide and tetracycline antimicrobials in groundwater and surface water using solid-phase extraction and liquid chromatography/mass spectrometry. Anal Chem.

[CR27] Martins AC, Pezoti O, Cazetta AL, Bedin KC, Yamazaki DAS, Bandoch GFG (2015). Removal of tetracycline by NaOH-activated carbon produced from macadamia nut shells: kinetic and equilibrium studies. Chem Eng J.

[CR28] Moussavi G, Heidarizad M (2010). Biodegradation of mixture of phenol and formaldehyde in wastewater using a single-basin MSCR process. J Bacteriol Res.

[CR29] Moussavi G, Heidarizad M (2011). The performance of SBR, SCR, and MSCR for simultaneous biodegradation of high concentrations of formaldehyde and ammonia. Sep Purif Technol.

[CR30] Moussavi G, Mahmoudi M, Barikbin B (2009). Biological removal of phenol from strong wastewaters using a novel MSBR. Water Res.

[CR31] Park H (2012). Reduction of antibiotics using microorganisms containing glutathione S-transferases under immobilized conditions. Environ Toxicol Pharmacol.

[CR32] Park H, Choung Y-K (2007). Degradation of antibiotics (tetracycline, sulfathiazole, ampicillin) using enzymes of glutathion s-transferase. Hum Ecol Risk Assess.

[CR33] Polesel F, Andersen HR, Trapp S, Plósz BG (2016). Removal of antibiotics in biological wastewater treatment systems—a critical assessment using the activated sludge modeling framework for xenobiotics (ASM-X). Environ Sci Technol.

[CR34] Rezaei R, Aghapour AA, Khorsandi H (2022). Investigating the biological degradation of the drug β-blocker atenolol from wastewater using the SBR. Biodegradation.

[CR35] Shao S, Wu X (2020). Microbial degradation of tetracycline in the aquatic environment: a review. Crit Rev Biotechnol.

[CR36] Shi Y-J, Wang X-H, Qi Z, Diao M-H, Gao M-M, Xing S-F (2011). Sorption and biodegradation of tetracycline by nitrifying granules and the toxicity of tetracycline on granules. J Hazard Mater.

[CR37] Suen WC, Spain JC (1993). Cloning and characterization of *Pseudomonas* sp. strain DNT genes for 2,4-dinitrotoluene degradation. J Bacteriol Res.

[CR38] Tao Y, Wang F, Meng L, Guo Y, Han M, Li J (2017). Biological decolorization and degradation of malachite green by *Pseudomonas* sp. YB: process optimization and biodegradation pathway. Curr Microbiol.

[CR39] Tchbanoglous G, Burton FL, Stensel HD (2003). Wastewater engineering: treatment and reuse.

[CR40] Weisburg WG, Barns SM, Pelletier DA, Lane DJ (1991). 16S ribosomal DNA amplification for phylogenetic study. J Bacteriol Res.

[CR41] Wiggins BA, Jones SH, Alexander M (1987). Explanations for the acclimation period preceding the mineralization of organic chemicals in aquatic environments. Appl Environ Microbiol.

[CR42] Wu J, Jiang Y, Zha L, Ye Z, Zhou Z, Ye J (2010). Tetracycline degradation by ozonation, and evaluation of biodegradability and toxicity of ozonation byproducts. Can J Civ Eng.

[CR43] Wu X, Wu X, Shen L, Li J, Yu R, Liu Y (2019). Whole genome sequencing and comparative genomics analyses of *Pandoraea* sp. XY-2, a new species capable of biodegrade tetracycline. Front Microbiol.

[CR44] Xiong H, Zou D, Zhou D, Dong S, Wang J, Rittmann BE (2017). Enhancing degradation and mineralization of tetracycline using intimately coupled photocatalysis and biodegradation (ICPB). Chem Eng J.

[CR45] Yahiat S, Fourcade F, Brosillon S, Amrane A (2011). Removal of antibiotics by an integrated process coupling photocatalysis and biological treatment—ase of tetracycline and tylosin. Int Biodeterior Biodegrad.

[CR46] Yusoff N, Ong S-A, Ho L-N, Wong Y-S, Mohd Saad FN, Khalik W (2016). Evaluation of biodegradation process: comparative study between suspended and hybrid microorganism growth system in sequencing batch reactor (SBR) for removal of phenol. Biochem Eng J.

[CR47] Zhang L, Song X, Liu X, Yang L, Pan F, Lv J (2011). Studies on the removal of tetracycline by multi-walled carbon nanotubes. Chem Eng J.

[CR48] Zhang D, Yin J, Zhao J, Zhu H, Wang C (2015). Adsorption and removal of tetracycline from water by petroleum coke-derived highly porous activated carbon. J Environ Chem Eng.

